# Contemporary male slings for stress urinary incontinence: advances in device technology and refinements in surgical techniques

**DOI:** 10.1177/17562872231187199

**Published:** 2023-07-25

**Authors:** Eric Chung

**Affiliations:** AndroUrology Centre, Suite 3, 530 Boundary Street, Brisbane, QLD 4000, Australia; Department of Urology, Princess Alexandra Hospital, University of Queensland, Brisbane, QLD, Australia; Macquarie University Hospital, Sydney, NSW, Australia

**Keywords:** clinical outcomes, male sling, scientific advances, stress incontinence, surgery

## Abstract

Synthetic male sling (MS) is considered an effective surgical treatment to restore male stress urinary incontinence. The modern MS can be categorised into adjustable or non-adjustable types, while the surgical techniques can be divided into retropubic or transobturator approaches. This narrative review paper evaluates the contemporary MS devices in the current commercial market regarding clinical outcomes and refinements in surgical techniques. Scientific advances in device design and technology, coupled with further surgical refinements will enhance the clinical outcomes and improve the safety profile of MS surgery. The newer generation of modern MS not only provides direct compression of the bulbar urethra but also allows for proximal urethral relocation by realigning the mobile sphincter complex to provide further urethral sphincter complex coaptation. Strict patient selection, use of MS with proven clinical records, adherence to safe surgical principles and judicious postoperative care are critical to ensure a high continence rate, good patient satisfaction and low postoperative complications.

## Introduction

Synthetic male sling (MS) is considered an effective surgical treatment to restore male stress urinary incontinence (SUI). From the early days of the male bulbourethral sling to the current modern MS, there have been considerable advances in device design, technology and surgical techniques.^1^ The objective of the MS is to provide a direct compression of the bulbourethral in a hammock-like effect and result in urethral luminal coaptation. Further improvement in our understanding of the male continence mechanism and lessons learned from earlier models of MS have resulted in better sling materials and refined surgical techniques, which translates to better clinical outcomes and safety profiles for males with SUI.^2^ The ideal surgical candidate should have a mild to moderate degree of SUI with an estimated 24-h pad weight of less than 500 g at least 6 months following radical prostatectomy and possesses a reasonable residual urinary sphincteric function.^3,4^ The patient will need to be able to generate sufficient detrusor contraction to overcome the fixed resistance of the sling to void spontaneously and remain dry with physical activity due to urethral coaptation. The proposed advantages of MS include its relatively low cost, less invasive nature and relatively simple procedure, and that patients can void spontaneously.^1,2,5^

The modern MS can be categorised into adjustable or non-adjustable types, while the surgical techniques can be divided into retropubic or transobturator (TO) approaches. Commercially available adjustable MS include Argus (Promedon, Cordoba, Argentina), ReMeex (Neomedic, Barcelona, Spain) and ATOMS (AMI, Feldkirch, Austria), while the current non-adjustable MS are AdVance (Boston Scientific, Minnetonka, USA), Virtue (Coloplast, Minneapolis, USA), I-STOP TOMS (CL Medical, France) and Surgimesh M-SLING (Aspide Medical, France) slings. The adjustable MS has a theoretical benefit over non-adjustable MS as the sling can be revised (adjusted) to provide further urethral compression in the event of persistent and/or recurrent urinary incontinence.^1^

While there are very few studies that directly compared the adjustable and nonadjustable MS, the overall clinical efficacy of adjustable MS does not appear to support the mechanical adjustability of such MS to have better continence outcomes when compared to a fixed MS if the sling was placed with proper tension and/or had adequate urethral coaptation.^1–3^ Nonetheless, the idea of being to restore or improving continence with an adjustable MS is appealing to many patients. Chung *et al*.^6^ reported that more men chose adjustable over non-adjustable MS when given the options despite no significant difference observed in the clinical outcomes and similar patient satisfaction rates.

This article aims to provide a narrative review of the contemporary MS devices in the current commercial market and evaluates current refinements in surgical techniques and limitations related to MS (Table 1).

**Table 1.** Comparison of various male slings in the commercial market.

Name	Company	Device specification	Unique features
Argus	Promedon	Argus and Argus-T A silicone foam pad connected to two silicone (multiple cone-like sections) columns and 2 silicone rings/washers	Adjustable sling Intra-operative tensioning based on retrograde leak point pressure (60 cmH_2_O in virgin and 40–50 cmH_2_O in revision cases
Remeex	Neomedic	Polypropylene sling with two monofilament traction threads on both ends	Adjustable sling Connection and adjustment with a central mechanical regulator
AdVance	Boston Scientific	AdVance and AdVance XP Polypropylene material with 2 mesh arms	Non-adjustable sling AdVance XP incorporates suture thread across the sling material, chevron anchors, and a different trocar configuration (compared to original AdVance)
Virtue	Coloplast	Quadratic monofilament central polypropylene mesh with four mesh arms	Non-adjustable sling Prepubic arms and central mesh can be fixed to pubic bone for further urethral compression
I-STOP TOMS	CL Medical	Monofilament polypropylene central mesh with four mesh arms	Non-adjustable sling Sling arms can be placed both outside-in or inside-out approach
Surgimesh M-SLING	Aspide Medical	Quadratic polypropylene central mesh with four mesh arms	Non-adjustable sling
ATOMS	AMI GmbH	A polypropylene mesh tape with an adjustable soft inflatable silicone cushion connected to a refillable titanium port	Adjustable sling Additional volume can be injected into the titanium port

## Materials and methods

This narrative review evaluates the contemporary MS devices currently marketed in the commercial market and serves as an extension to the recent 7th International Consultation on Incontinence report on male surgery for urinary incontinence chapter. Available literature about MS between 1 January 2000 and 1 July 2022, with the following keywords such as ‘male sling’, ‘male continence surgery’ and ‘stress urinary incontinence’ was reviewed on MEDLINE and EMBASE databases. This narrative review summarises pertinent clinical studies on these MS devices and published outcomes of interest may vary since some of the MS devices have a higher volume of publications, so the decision was taken that important articles, especially those considered major studies, were included only. When applicable, additional information regarding the nuances of surgery including refinements in surgical techniques for the reported MS devices is provided.

## Clinical findings

### Argus sling (Promedon, Cordoba, Argentina)

#### Manufacturer and device specifications

The Argus male sling was first released by Promedon in 2004 as a retropubic MS, while the newer version, the Argus-T which is inserted through a TO route was introduced several years later.^7^ The Argus adjustable suburethral sling has a silicone foam pad which is connected to two silicone columns made of multiple cone-like sections and two silicone rings/washers. The rings are positioned on the columns to regulate the tension of the silicone cushion on the bulbar urethra. The coned structure of the columns allows regulation of sling tension by tightening or releasing the two silicone rings.

#### Surgical techniques and clinical outcomes

The original Argus sling arms are placed in a retropubic approach while the newer Argus-T arms are performed through the TO route. To achieve satisfactory continence, the Argus sling is tensioned by pushing the silicone washers along the conical elements of the silicone columns, to a recommended maximum intra-operative retrograde leak point pressure between 40 and 50 cmH_2_O (which is lower than the traditional 60 cmH_2_O in virgin cases). For revision surgery to address recurrent SUI, the sling can be further tightened through a small non-invasive incision at each side of the sling arm where the silicone washers can be readjusted to provide further urethral compression.^8^ Comparing different surgical approaches, one study found higher continence rates (33.3% *versus* 11.8%, *p* = 0.114) with superior functional outcomes and lower explantation rates after Argus classic compared to Argus-T MS.^9^ Comparing inguinal-perineal incision to single-perineal incision for the Argus-T MS, one study^10^ found that the inguinal approach was associated with higher infective complications when compared to the single-incision approach.

Published literature showed the Argus MS to be relatively effective and many patients (up to a third) will require sling readjustment.^8–10^ However, complications such as sling erosion or infection are not uncommon and the device explantation rate remains quite high (up to 16%).^11–15^

### Remeex sling (Neomedic, Barcelona, Spain)

#### Manufacturer and device specifications

The Remeex sling, also known as the REadjustable MEchanical EXternal device, is manufactured by Neomedic in 2004.^16^ This adjustable MS system consists of a polypropylene suburethral sling with two monofilament traction threads on both ends for connection to a central mechanical regulator.

#### Surgical techniques and clinical outcomes

The Remeex sling is inserted in a retropubic approach with the polypropylene sling mesh placed in contact with the bulbocavernosus muscle and an additional transverse suprapubic incision to accommodate the traction threads. A mechanical regulator (known as a varitensor) adjusts the threads’ tension on the sling using an external manipulator until urinary continence is achieved.^17^

Published studies reported that Remeex MS achieves a reasonable continence rate (as high as 64.7%) although most patients required at least two adjustments.^18–20^ However, complications such as bladder perforation (up to 29%), urinary retention (up to 36%) and sling explantation (up to 21%) are not uncommon.

### AdVance sling (Boston Scientific, formerly American Medical Systems, MN, USA)

#### Manufacturer and device specifications

The AdVance MS is a non-adjustable male sling introduced by American Medical Systems (now Boston Scientific) around 2006. This male sling is made of polypropylene material with two (outside-in) arms and is inserted through a TO route. In 2010, the newer AdVance XP version was released and incorporates several unique features such as chevron anchors and different trocar sizes.^21^

#### Surgical techniques and clinical outcomes

The placement of the AdVance involves incising the bulbospongiosus muscle and transecting the central tendon so that the sling can be sutured to the bulbar urethra.^22^ When the sling is tensioned, the bulbar urethra is compressed and ‘repositioned’ proximally and caudally, meaning the sling is no longer visible on the perineal wound. The amount of AdVance sling tension is based on good proximal relocation of the sling and the presence of urethral coaptation under direct cystoscopy examination.^23^ Advances in design with AdVance XP MS incorporate thread across the sling material to avoid the roll-on effect, chevron anchors to limit sling slippage and a different trocar configuration to facilitate sling placement in bigger patients. There have been numerous publications on an AdVance sling over the years. Initially, the AdVance sling was tensioned based on retrograde leak point pressures >60 cmH_2_O,^24^ but recent modifications include mandatory cystoscopy evaluation, the passage of sling at the apex of the ischiopubic rami and newer AdVance-XP sling.^25–28^

Published literature reported an initial continence rate of up to 87.3% but a gradual decline down to under 60% at 5-year follow-up period.^22,29–35^ More recently, one study reported that the AdVance male sling appears to improve male sexual function with regards to erectile and orgasm domains in addition to urinary continence.^36^

Reported adverse events are generally mild with transient urinary retention and groyne pain being the most frequently encountered, and the need for explantation is exceedingly low.^37^ Patients with severe incontinence (>400 g/24-h pad weight), a history of prior radiotherapy or coexisting storage dysfunction are more likely to have poorer outcomes.^1,29,22^ Failure of the AdVance sling can be salvaged with an AUS or similar devices^38^ since a secondary sling procedure is not suitable and has a poor success rate.^39^

### Virtue sling (Coloplast, Humlebaek, Denmark)

#### Manufacturer and device specifications

The Virtue MS is a quadratic non-adjustable MS manufactured by Coloplast and introduced in 2009.^40^ This quadratic urethral sling consists of monofilament polypropylene mesh (5.5 cm × 7 cm) with four mesh arms, two (inside-out) TO arms and two (outside-in) prepubic arms and is designed to provide both urethral relocation (TO arms) and ventral elevation (prepubic arms).

#### Surgical techniques and clinical outcomes

In contrast to the AdVance outside-in TO approach, the Virtue sling is placed using the J-hook passer inside-out through the TO space while the prepubic arms passed from the perineal surgical wound to suprapubic skin.^41,42^ Further modifications to the surgical technique include tensioning of the TO arm laterally to elevate the bulbous urethra and the prepubic arms superiorly similarly to compress the bulbous urethra, re-routing the TO sling arms back into the perineal wound to be fixed to their opposite side to secure the mesh in place, and additional fixation sutures can be placed onto the prepubic mesh arms to the underlying pubic bone to provide further distal urethral compression, similar to the InVance sling mechanism of action.^43,44^ Improper fixation of the arms can result in a substantially lower success rate compared to a sling with properly fixated prepubic and TO sling components.^43^ In terms of revision or salvage surgery, imbricating non-absorbable sutures within the existing mesh and further tensioning based on retrograde leak point pressure may improve the continence rate.^44^

Published literature showed a reasonable continence rate greater than 70%.^30,45–47^ Like the AdVance sling, greater severity of incontinence, older males and previous radiation therapy are associated with higher postoperative incontinence.^1,2^ Common postoperative complications include wound infection, perineal pain, and urinary retention although the incidence of pubic bone osteitis as seen with the InVance MS has not been reported in the literature.

### I-STOP TOMS sling (CL Medical, France)

#### Manufacturer and device specifications

The I-STOP TOMS sling was developed by CL Medical in 2009.^48^ The development of this MS is thought to arise from the female I-STOP TO sling and consists of a monofilament polypropylene 4-arms sling (two arms on each side) measuring 4.5 cm × 1.4 cm, with a 2.8-cm central part placed over the urethra.

#### Surgical techniques and clinical outcomes

This MS is placed through a TO approach, but the central tendon is not divided. The sling allows for either an outside-in or inside-out approach with the helical needle used along with blunt dissection to guide the passage through the TO space for the passage of the two arms on each side. The sling is tensioned following sutures to the bulbar urethral. The utilisation of four arms ISTOP TOMS appeared to produce higher continence outcomes.^49^ The larger surface area was proposed in addition to the fact that the sling is designed to be placed more distally, over the bulbar and post-bulbar urethra, which has bulbospongiosus coverage, which would reduce the risk of erosion.^49^

To date, published data on ISTOP-TOMS showed around 60% of patients achieved social continence although its efficacy dramatically decreased to 20% at 5 years.^50–54^ Nonetheless, device failure is not uncommon (reported in more than a third of cases) with recurrent incontinence.^54^

### Surgimesh M-SLING (Aspide Medical, France)

#### Manufacturer and device specifications

The non-adjustable Surgimesh M-SLING is produced by the Aspide Medical around the mid-2010s.^55^ This quadratic four-arm polypropylene urethral sling consisted of TO and prepubic arms, similar to the Virtue MS.

#### Surgical techniques and clinical outcomes

Like the placement of Virtue MS, this sling was designed for direct implantation over the urethral bulb and includes two TO and two prepubic arms with divergent traction axes to provide adequate. So far, only a single report can be found on this device with reported total continence of 34.4% although more than two-thirds (71%) of patients were satisfied at 24 months follow-up.^56^ However, no further information can be obtained whether this device has been discontinued or withdrawn from the commercial market.

### ATOMS sling (A.M.I. GmbH, Feldkirch, Austria)

#### Manufacturer and device specifications

The adjustable TO male system (ATOMS) sling is developed and marketed by Agency for Medical Innovations (AMI) in 2010.^57^ The ATOMS system composes of a TO-placed polypropylene mesh tape with an adjustable soft inflatable silicone cushion, connected to a refillable titanium port. The original model has an inguinal port while subsequent designs have used a scrotal port.

#### Surgical techniques and clinical outcomes

The surgical technique is similar to any outside-in TO sling. The two mesh arms of the ATOMS device are secured in place following rerouting on either side through the obturator foramen to attach back onto the central cushion component, thereby creating a firm, four points fixation point. The implant is connected by a catheter to a titanium port, which is placed in the scrotum. This allows for the system’s pressure postoperatively adjustment by altering the filling volume of the cushion. Further adjustment can be carried out every 6 weeks to ensure good continence.

Published studies showed close to two-thirds of patients were dry^58–60^ with the mean adjustments per patient expected up to four times.^61,62^ However, device removal following infection is not uncommon and has been reported to be as high as a third of cases (30,6%).^63^ The pre-connected scrotal port had the lowest complication rate with device explantation compared to the earlier inguinal port group (24/30 cases *versus* 30/37 cases).^62^ Other complications such as titanium intolerance (41% of patients) and device leakage (21% of devices) at the port or cushion have also been documented.^64^

## Discussion

The MS is generally advocated to treat patients with mild to moderate incontinence, and ideally in those without prior history of radiation and have residual sphincter activity for better urethral coaptation.^1,2^ While adjustable MS has the theoretical advantage of restoration of continence with minor ‘readjustment’ either by increasing sling tension or fluid within the compressing urethral pad, they are associated with potentially higher complication rates and, at the end of the day, may not reciprocate the initial continence outcome seen in non-adjustable MS.^65^

Advances in sling design and technology coupled with further refinements in surgical technique have improved each sling outcome and safety profile. However, there is no conclusive evidence to suggest that one type of MS is better than another, and there is no evidence that the adjustability of the MS offers an additional benefit over other types of slings with regards to safety profile in the longer-term. A direct comparison between various MS devices in terms of superiority of continence is difficult and probably not accurate given the heterogeneous study population, variable definitions of incontinence before MS surgery and characterizations of ‘cure’ or ‘improvement’ following treatment, as well as the inconsistent use of validated and non-validated outcome measures and lack of blind randomisation.

The recent publication of the non-inferiority MASTER clinical trial^66^ comparing MS surgery to AUS in patients with any degree of SUI who are suitable for surgery found that MS has similar continence outcomes to the AUS [difference 3.6% (95% CI −11.6 to 4.6, *P*_NI_ = 0.003)], with incontinence symptoms (ICIQ-UI SF) reduced from scores of 16.1 and 16.4 at baseline to 8.7 and 7.5 for MS and AUS, respectively [mean difference 1.4 (95% CI 0.2–2.6), *p* = 0.02]. Nonetheless, all secondary and post hoc analyses were in favour of an artificial urinary sphincter than MS. Current evidence showed that certain patient factors are associated with higher sling surgery failure or complication rates such as more severe preoperative incontinence, the presence of urethra stricture, pelvic radiation, the presence of neurological disorder, de-novo storage symptoms, poor native sphincteric activity, advanced age and need for on-going endoscopic procedures.^67–71^ The presence of intraoperative findings such as inadequate urethral coaptation and lack of proximal bulbar urethral compression or relocation could signify fewer effective outcomes.^1,72^ It is important to counsel patients that an improvement in continence can be expected with MS surgery, but there is no guarantee that one will be completely continent, and recurrence of SUI invariably will occur sometime in the future.

While MS is a minimally invasive surgery that can instantaneously restore urinary continence with the patient avoiding the need to manipulate a device to void (as in AUS), it is not without limitations and surgical risks.^1,2^ Postoperative pain such as discomfort in the inner legs is not uncommon, while contemporary literature reports a postoperative continence rate of around 70–75%.^3–5^ Furthermore, patients with a history of pelvic radiation or trauma may not be suitable to have MS surgery.^3,4^ For those with persistent or recurrent SUI following non-adjustable MS surgery, it is generally not advisable to perform another MS to salvage these cases, with an AUS being the preferred surgical solution.^1,3^ Nonetheless, despite the limitations mentioned above, MS has an important role in post-prostatectomy incontinence and is effective if offered to the suitable surgical candidate following comprehensive evaluation and proper informed consent. Future directions in research and design development will likely incorporate higher-grade synthetic materials that are more robust to provide better urethral coaptation, better safety profile, and perhaps antibiotic-eluted properties to minimise infection risk, as well as an easier surgical insertion with additional tools tailored for specific body habitus.

## Conclusion

Scientific advances in device design and technology, coupled with further refinements in surgical techniques will enhance the clinical outcomes and improve the safety profile of MS surgery. The newer generation of modern MS not only provides direct compression of the bulbar urethra but also allows for proximal urethral relocation by realigning the mobile sphincter complex to provide further urethral sphincter complex coaptation. Strict patient selection, use of MS with proven clinical records, adherence to safe surgical principles and judicious postoperative care are critical to ensure a high continence rate, good patient satisfaction and low postoperative complications.

## Declarations

**Ethics approval and consent to participate:** Not applicable.

**Consent for publication:** Not applicable.

**Availability of data and materials:** Not applicable.
